# Label Metric for Multi-Class Multi-Target Tracking under Hierarchical Multilevel Classification

**DOI:** 10.3390/s22228613

**Published:** 2022-11-08

**Authors:** Jingdong Diao, Qingrui Zhou, Hui Wang, Ying Yang

**Affiliations:** Qian Xuesen Laboratory of Space Technology, China Academy of Space Technology, Beijing 100094, China

**Keywords:** multi-target tracking, hierarchical multilevel classification, OSPA metric, performance evaluation

## Abstract

Aiming at multiple quantities and types of targets, multi-class multi-target tracking usually faces not only cardinality errors, but also mis-classification problems. Considering its performance evaluation, the traditional optimal subpattern assignment (OSPA) method tends to calculate a separate metric for each class of targets, or introduce other indexes such as the classification error rate, which decreases the value of OSPA as a comprehensive single metric. This paper proposed a hierarchical multi-level class label for multi-class multi-target tracking under hierarchical multilevel classification, which can synthetically measure the state errors, cardinality error, and mis-classification. The hierarchical multi-level class label is introduced as an attached label to finite sets based on the hierarchical tree-structured categorization. A Wasserstein distance type metric then can be defined among the distribution represented by any two labels. The proposed label metric is a mathematic metric, and its advantages are illustrated by examples in several cases.

## 1. Introduction

Multi-target tracking (MTT) with multiple heterogeneous sensors has a wide range of applications in the fields of autonomous driving, surveillance in maritime and aerial space, and so on. Just like the metrics on vectors such as Euclidean distance and Mahalanobis distance, which represent the meaning of the miss-distance between two states of object in single-target tracking, a definition of metric between two finite sets is also of importance in multi-target state tracking. The concept of the metric herein stands for a distance function on finite sets, which satisfies non-negativity, symmetry, identity of indiscernibles and triangle inequality in a mathematical sense. In the problem of MTT, a well-defined metric is significant in following aspects: (1) Performance evaluation. Metrics can give a mathematically consistent miss-distance between estimates and the ground truth to evaluate algorithm performance [1]. (2) Estimate criterion. Metrics can also be considered as a criterion to obtain estimators from the posterior probability density of random finite sets, for example, minimizing the mean optimal subpattern assignment (OSPA) [2,3,4,5]. (3) Sensor management. Metrics for finite sets has potential applications in multisensor-multitarget sensor field of view (FOV) management, since its many-to-many consistency in optimizing [6,7]. (4) Definition of metric space. Relying on the metric space derived from the definition of metric on a space of finite sets, one can rigorously analyze the convergency of estimators, or do a nearest neighbor search, cluster and classify on finite sets [8,9,10].

The performance evaluation of MTT is a long-standing problem across many topics. Both metrics and non-metrics have been discussed [6,11,12,13,14,15]. Considering the indispensable importance of metrics’ mathematically consistency in performance evaluation, pointed out in [1], here we focus on metrics and briefly review several widely used ones in MTT. The Hausdorff distance on finite sets is the first metric applied in MTT. Due to the insensitivity to differences in cardinalities of finite sets and overreaction to outliers with the Hausdorff distance, the optimal mass transfer (OMAT) metric was proposed in [6], bases on the Wasserstein distance between the distributions of finite sets. However, simply by normalizing the unbalanced cardinalities to construct a distribution leads to counterintuitive results sometimes with the OMAT metric. Proposed by [11], OSPA balanced the difference of cardinalities by bring “dummy” point into finite sets, then bounded the penalty of cardinality; as a consequence, it is more intuitive than the former ones. On this basis, Generalized OSPA (GOSPA) [12] and Complete OSPA (COSPA) [16] was proposed to avoid the “spooky effect” in optimal OSPA estimation and completed it [17]. Several patches based on OSPA have been put forward as well, such as Hellinger-OSPA [18], which concerned the uncertainty, Q-OSPA [19], which add a quality factor to OSPA, Multi-Group OSPA [20] for hierarchical finite sets, and IoU-OSPA [1], which adapted for the bounding boxes in vision tasks. Because of the effectiveness of OSPA, the metrics on the space of finite sets of trajectories [21,22,23,24] and metrics utilized for SLAM [25], etc., emerged, which enrich the application range of the metrics.

As a resent development in deep learning, edge computing and sensor technology, in both the demand and approach side, the MTT problem tends to integrate the complexity with multi-class information from multiple heterogeneous sensors. On one hand, scenes increasingly diversified, such as fusing multiple views of vision and V2X information to track cars, cyclists and pedestrians at the crossroads, or tracking all kinds of ships with the optical/SAR image and AIS information in the space-based maritime surveillance. One common characteristic of the aforementioned scenes is that the information is collected from different sensors, which has multiple levels of class labels. For example, V2X and AIS messages from cooperative targets always have ID numbers to distinguish individuals, e.g., MMSI for ships, which is precise information belonging to the individual level. Meanwhile, information detected and classified from the optical/SAR image is much less precise, which, divided into different levels of classes, depends on the resolution and classifier itself. On the other hand, joint tracking and classification approaches [26,27] have gained more attention. All these motivations require a metric that can evaluate the miss-distance between finite sets with both state and class information in a unified way, and also conform to a meaningful interpretation that captures both cardinality errors, state errors, and mis-classification. However, as far as the authors’ knowledge, there is no proposed metric yet which is mathematically rigorous and satisfies all above-mentioned requirements. In [6], a heuristics extension of OMAT is mentioned on space (x,c) including target kinematic states *x* and class *c*, whose ground distance is $d\left(\left(x_{1},c_{1}\right),\left(x_{2},c_{2}\right)\right)=\sqrt{∥x_{1}-x_{2}{∥}^{2}+{∥\pi \left(c_{1}\right)-\pi \left(c_{2}\right)∥}^{2}}$, where π(c) represents a certain point in a Euclidean space associated with class *c*. However, this metric was neither further studied, nor can be used for handling multi-level class labels. In addition, paper [28] attempted to introduce a joint probability divergence (JPD) to quantify tracking error, mis-classification, and their interdependence. However, it is not an applicable metric either.

Aiming at the above problem, this paper proposed a hierarchical multi-level class label for multi-class multi-target tracking under the hierarchical multilevel classification, which can synthetically measure state errors, cardinality error, and mis-classification. The hierarchical multi-level class label is first introduced as an attached label to finite sets based on the hierarchical tree-structured categorization. A Wasserstein distance type metric then can be defined among the distribution represented by any two labels. The proposed label metric is mathematically metric, and its advantages are illustrated by examples in several cases.

The contributions of this paper include:

(1) Proposed one kind of multilevel class label based on the hierarchical tree structured category, which enhanced finite sets with a label that can completely cover the space of multi-class multi-target tracking problems.
(2) Proposed and proved a mathematically metric of the aforementioned hierarchical multilevel class label, and extended the traditional OSPA metric as a new metric of finite sets with the hierarchical multilevel class label, which can serve as the foundation for later research on tracking algorithms.
(3) Several cases are also given to illustrate features and advantages of the proposed metric.

The rest of this paper is organized as follows. Section 2 reviews how to represent multi-target states with finite sets, reminds the three axioms of metrics, as well as the definitions of de facto standard metrics including OSPA and GOSPA. Motivated by better describing the state of multi-class multi-target systems, we proposed a definition of the class label called the HMC label, with its Wasserstein distance type metric in Section 3, and proved that it is a truly metric in the set-valued label space. Finally, the aforementioned extended OSPA-type metrics are illustrated by several numerical cases in Section 4, and Section 5 gives the conclusion.

## 2. Background of Metrics for Multi-Target Systems

This section recalls some necessary background on multi-target systems’ states, which are represented by finite sets, the definition of its metrics, the OSPA metric for multi-target systems, and its extensions.

Since the study object of multi-target tracking is no longer a single dynamic object, but a variable number of objects, correspondingly, the mathematical element describing its state naturally expands from a vector of real numbers to a finite set. For more rigorous theory, please refer to Mahler’s book [29].

Assume that the real number vector $x\in X\subseteq R^{n}$ represents the state of a target. The finite set $X=\{x_{1},\ldots ,x_{n}\}$ is defined as a set of finite numbers of vectors, which corresponds to the same number of targets. *n* is called the cardinality of the finite set *X*, and X∈F(X), where F(X) is the set of all subsets of single state space X.

**Definition 1** (Metric of finite sets). *Let* X,Y,Z∈F(X) *be arbitrary finite sets with arbitrary cardinalities. A function* $d:F\left(X\right)\times F\left(X\right)\to R_{+}=\left[0,\infty \right)$ *is called a metric of finite sets if it satisfies the following three axioms:*

*(1)*  *(Identity):*d(X,Y)=0*if and only if*X=Y;
*(2)*  *(Symmetry):*d(X,Y)=d(Y,X)*for all*X,Y∈F(X);
*(3)*  *(Triangle inequality):*d(X,Y)≤d(X,Z)+d(Z,Y)*for all*X,Y,Z∈F(X).

Early metrics includes Hausdorff distance of finite sets and optimal mass transfer (OMAT) metric [6]. However, due to their respective shortcomings in characterizing the mis-distance between finite sets, currently the commonly used metric is OSPA [11], and GOSPA [12], etc.

## 3. Hierarchical Multi-Level Class Label and Its Metric

In this section, a definition of a kind of finite discrete state called the hierarchical multi-level class label is first introduced, which denotes the classification label of a single target sample in a hierarchical structured category tree. Then, a metric applying to hierarchical multi-level class labels is given based on the Wasserstein distance and a corresponding definition of ground distance.

### 3.1. Definition of Hierarchical Multi-Level Class Label

As the variety of sensors increases and the price decreases, more multi-target tracking solutions tend to use multiple and diverse sensors to accomplish their tasks. For example, in the automatic driving scenario, vehicles combine cameras, LiDAR, and radars to detect and track cars, pedestrians, and cyclists on open roads; Another example is tracking ships by fusing AIS and SAR/optical images from satellites in maritime surveillance tasks. The common ground of all these examples is that the information given by diverse sensors is belonging to multi-level categories, due to their different capabilities and resolutions, which can be attached a label to provide a hypothesis on the identity of the targets. These labels are usually hierarchically multi-level structured. The North American Treaty Organization (NATO) AAP-6 Glossary has provided a precise definition of terminology used for target classification, which is viewed as a hierarchy [30], in which the targets are categorized into more and more precise subclasses, from detection to fingerprinting (coarsest to most precise). For example, in maritime surveillance, a low-resolution SAR satellite may only offer us the coarsest detection-level information, which can only indicate a ship existing there. As the resolution increases, information will be more precise, so that one can classify a target. This is until the individual-level, where the information was provided from the cooperative target by AIS, for instance.

With the increasing prevalence of hierarchical multi-level class information fusion, it is necessary to incorporate it into a unified RFS space and propose a metric for it as well. A hierarchical multi-level class label is a hypothesis of the target identity based on observed data, where the target identity can be viewed as a parameter (or state) of the target on a finite discrete space. When the sensor does not have the ability to distinguish the precise identity of the target, it can only give a coarser guess of it. Essentially, hierarchical multi-level class labels semantically represent a set of identities with uncertainty, hence they can be observed as set-valued observations of identity, i.e., the identity of target is observed, but the result can only indicate to which set the target belongs. Here, it gives its definition and probability model, as follows:

Consider a multi-class multi-target system with a specific identity formalized as a labeled finite set

$$X=\left\{\left(x_{1},c_{1}\right),\ldots ,\left(x_{\left|X\right|},c_{\left|X\right|}\right)\right\}\in F\left(R^{n}\times C\right),$$

where |X| is the cardinality of the finite set *X*, i.e., the number of targets. Let the Cartesian product $R^{n}\times C$ be the single-target state space, i.e., $\left(x,c\right)\in R^{n}\times C$, where vector $x\in X\subseteq R^{n}$ is the state, and the so-called individual-level label c∈C represents the unique identity of the target taking values from a discrete finite space, such as $Z_{+}$. The observation of the multi-target system is also denoted by a finite set

$$\overset{ˇ}{X}=\left\{\left(x_{1},C_{1}\right),\ldots ,\left(x_{\left|X\right|},C_{\left|X\right|}\right)\right\}\in F\left(R^{n}\times F\left(C\right)\right),$$

where the only difference is the label C∈F(C) which here is extended to the hierarchical multi-level class label.

**Figure 1:**
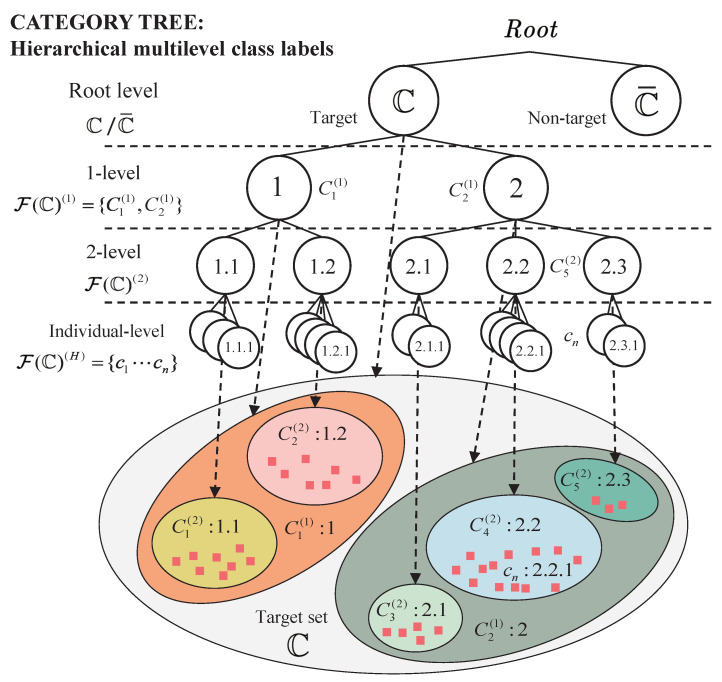
The category tree.

As illustrated in Figure 1, the hierarchical multi-level class label is defined as a tree-structured hierarchy [31,32,33]. First, on the “root” level, all the original signals or image boxes are separated into the target set C or non-target set $\bar{C}$. This process is also known as detection, in which interest targets are distinguished from other clutters, e.g., background, noise and unfocused objects. Then, the target set C is hierarchically partitioned into several subset in multi-levels until the individual-level. Every label denotes a set partition from a finite target set. Let the level of category, i.e., the depth of the category tree, be h=1,…,H. Denote a *h*-level class label as $C^{\left(h\right)}$, which represents a set taking from $F{\left(C\right)}^{\left(h\right)}\subset F\left(C\right)$. Define the aforesaid sets *C*, satisfying the following three axioms:

(1) (“IS-A” relationship): All class concepts represented by label *C* have an asymmetric, anti-reflexive, and transitive class taxonomy relationship; refer to [32];
(2) (Non-overlapping): For arbitrary two sets on same level, $\forall C_{1}^{\left(h\right)},C_{2}^{\left(h\right)}\in F{\left(C\right)}^{\left(h\right)}$, $C_{1}^{\left(h\right)}\cap C_{2}^{\left(h\right)}=⌀$;
(3) (Full coverage): For arbitrary set represented by *h*-level label $C^{\left(h\right)}$, its next level subsets satisfy ${⋃}_{C^{\left(h+1\right)}\subset C^{\left(h\right)}}C^{\left(h+1\right)}=C^{\left(h\right)}$; Moreover, for all sets represented by *h*-level label $C^{\left(h\right)}$, ${⋃}_{C^{\left(h\right)}\in F{\left(C\right)}^{\left(h\right)}}C^{\left(h\right)}=C$.

### 3.2. Metric between Hierarchical Multilevel Class Labels

In this section, we first propose a metric to evaluate the miss-distance between hierarchical multilevel class labels, as a basis for extending the OSPA metric.

As mentioned above, a class label represents a finite set of discrete individual labels. It is essentially a set-valued discrete variable. Therefore, the Wasserstein distance can be naturally applied to this case.

**Assumption 1** (Ground distance of individual-level labels). *There exists a definition of the ground distance for individual-level labels, denoting as* $d_{g}\left(\cdot \right)$*, a metric that satisfies both intuitive and practical application requirements.*

In general, this ground distance indicates a penalty for mistaken identity. If switching identities would be costly, the distance between the two labels should be chosen to be greater than the inessential ones. To some extent, heuristics are inevitable in the implementation process [6]. However, there are still ways to ensure that the ground distance between the individual-level labels meets the axiomatic requirements of metrics. One intuitive approach is to embed all individual-level labels in a Euclidean space, and then use the metric between the associated points as the distance of the labels. Another practical approach is multi-step embedding, which will be demonstrated later. In this approach, the first step is to embed the high-level class into a Euclidean space, then each individual-level label in a same class is associated with the vertex of a simplex in the extended dimension.

Assume the target we concerned has a blank probability distribution of the element class, called ground distribution. Denote it as a histogram (or probability vector) $p_{C}\in \Sigma_{\left|C\right|}$ that belongs to the probability simplex

$$\Sigma_{\left|C\right|}\overset{def.}{=}\left\{p_{C}\in R_{+}^{\left|C\right|}:\sum_{i=1}^{\left|C\right|}p_{c_{i}}=1\right\}.$$

The discrete probability measure of the hierarchical multilevel class label *C* is denoted as

(1) $$\zeta_{C}=\frac{1}{\underset{c_{i}\in C}{\sum }p_{c_{i}}}\sum_{c_{i}\in C}p_{c_{i}}\delta_{c_{i}},$$

where $\delta_{c_{i}}$ is the Dirac delta function on its elements, an individual-level label $c_{i}$. The histogram of *C* can be expressed as a vector $p_{C}$,

$$p_{C}=\frac{p_{C}{1\;1}_{C}}{\underset{c_{i}\in C}{\sum }p_{c_{i}}},$$

where diagonal matrix ${1\;1}_{C}$ is a indicator, i.e., if $c_{i}\in C$, ${1\;1}_{Ci,i}=1$; otherwise, ${1\;1}_{Ci,i}=0$.

**Definition 2** (HMC-distance). *Let $C_{1}=\left\{c_{1},\ldots ,c_{m}\right\}$ and $C_{2}=\left\{c_{1}^{\prime },\ldots ,c_{n}^{\prime }\right\}$ be the arbitrary two hierarchical multilevel class labels on a category tree, i.e., $C_{1},C_{2}\in T\left(C\right)\subset F\left(C\right)$. Their discrete probability measure defined by (1) are $\zeta_{C_{1}},\zeta_{C_{2}}$ with histogram vector $p_{C_{1}},p_{C_{2}}$, respectively. Given the metric space $C,d_{g}$, where C is the individual-level label space, $d_{g}$ is the ground distance given by Assumption 1. The HMC-distance of hierarchical multilevel class labels $d_{\mathrm{HMC}}\left(\zeta_{C_{1}},\zeta_{C_{2}}\right)$ is defined as the 1-th Wasserstein distance herein,*

(2) $$d_{\mathrm{HMC}}\left(\zeta_{C_{1}},\zeta_{C_{2}}\right):=\underset{t\in T(p_{C_{1}},p_{C_{2}})}{\inf}\left\{\sum_{i=1}^{m}\sum_{j=1}^{n}t_{i,j}d_{g}\left(c_{i},c_{j}\right)\right\},$$

*where the infimum is taken over all m×n transportation matrices in the set of valid transport plans $T(p_{C_{1}},p_{C_{2}})$,*

$$T\left(p_{C_{1}},p_{C_{2}}\right)=\left\{T\in R_{+}^{m\times n}:T1=p_{C_{1}},T^{T}1=p_{C_{2}}\right\}.$$

Denote the optimal transport plan as $t^{*}\in T^{*}\left(p_{C_{1}},p_{C_{2}}\right)$, then the HMC-distance can be written as

$$d_{\mathrm{HMC}}\left(\zeta_{C_{1}},\zeta_{C_{2}}\right)=\sum_{i=1}^{m}\sum_{j=1}^{n}t_{i,j}^{*}d_{g}\left(c_{i},c_{j}\right).$$

For the former definition, the proposed HMC-distance $d_{\mathrm{HMC}}\left(\cdot \right)$ in the label space can be regarded as a Wasserstein distance of discrete distributions. As proved in the book [34], Wasserstein distance is indeed a metric, which obtains Definition 1, if the ground distance is a metric. As a consequence, the ground distance $d_{g}\left(\cdot \right)$ of individual-level labels should be designed as a metric to satisfy the Assumption 1. Section 4 provides an example that individual-level labels can be embedded to a Euclidean space, where the position can be set up heuristically by the confusion cost between classes, and the base ground distance within can be assigned as a norm distance, which obtains metric axioms.

### 3.3. OSPA-Type Metrics Extended with HMC Labels

For OSPA-type metrics, such as basic normalized/unnormalized OSPA and GOSPA, ref. [11,12] have proved that they are mathematical metrics when the distance between the elements in two finite sets is selected as a metric. Usually, such a distance is chosen as the Euclidean distance. In fact, if this distance is another metric, the above inference also holds. For proof of the technique, please refer to [21], which has constructed an OSPA-type metric with a track label. In a similar way, OSPA-type metrics extended with proposed HMC labels can also be metrics, as long as the ground distance is constructed as a metric too. We give such an example in the following Section 4.

## 4. Example Cases

In this section, we give an example with several cases to illustrate the proposed hierarchical multilevel class labels, and its advantages when utilizing into practical applications.

**Figure 2:**
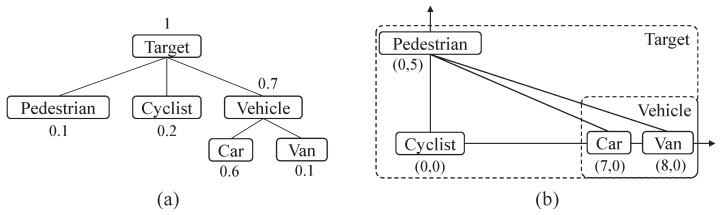
Diagram of example. (**a**) A simple category tree example with a root node “target” and two levels of classification labels. (**b**) The confusion costs between the individual labels “pedestrian”, “cyclist”, “car”, and “van”, embedded into a plane of Euclidean space.

Taking the autonomous driving scenario described in KITTI Benchmark as instance, consider the most common four kinds of class labels: “pedestrian”, “cyclist”, “car”, and “van”. As shown in Figure 2a, the object quantity of these four classes have a prior distribution of 0.1, 0.2, 0.6, and 0.1, respectively. With different sensors, the labels outputted by detection and classification algorithms may of a different level. Here, we provide a simple hierarchical multi-level category tree as the example: label “car” and “van” belonging to the label “vehicle”, and the root label named as “target”. Figure 2b shows how to embed four individual-level labels into a plane, and the position coordinates are marked in the figure. The ground distance is simply defined as the Euclidean distance to satisfy Assumption 1, and the HMC label metric then can be calculated by (2) of Definition 2, as shown in Table 1.

From Table 1, one can obtain that the cost of confusion between “pedestrian” and “car”/“van” is high, and as a result, the distance between “pedestrian” and the coarse classification label “vehicle” is also high. On the contrary, the confusion cost between “car” and “vehicle” is very low, which is consistent with intuition. This HMC label metric example can also be verified, which fulfills the requirements of Definition 1, especially the triangle inequality.

In this example, we also provide a selective case to illustrate the advantages of OSPA-type metrics extending with the former proposed definition of hierarchical multilevel class labels and its metric. Refer to [11], if the finite sets attached with hierarchical multilevel class labels, the ground distance d(x,y) in OSPA [11] should be replaced by

$$d\left(\left(x,C_{x}\right),\left(y,C_{y}\right)\right)={\left(d{\left(x,y\right)}^{p^{\prime }}+d_{\mathrm{HMC}}{\left(\zeta_{C_{x}},\zeta_{C_{y}}\right)}^{p^{\prime }}\right)}^{1/p^{\prime }}$$

and, in the same way, introduced by [21], where $1\leq p^{\prime }<\infty$, d(x,y) is a Euclidean distance, and $d_{\mathrm{HMC}}\left(\zeta_{C_{x}},\zeta_{C_{y}}\right)$ is the proposed HMC label metric by (2).

**Figure 3:**
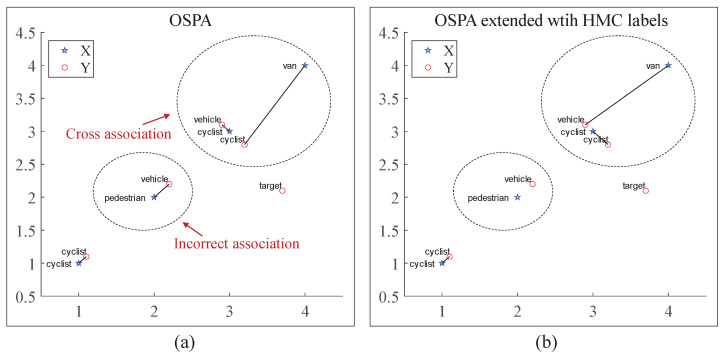
Association results in OSPA calculation step. (**a**) The association result with OSPA, where “X”, “Y” point sets stand for two finite sets. An incorrect association case and a cross-error case are circled. (**b**) The association result of OSPA-type metrics are extended with HMC labels.

Consider two HMC labeled finite sets, $\overset{ˇ}{X}$ and $\overset{ˇ}{Y}$; the positions and labels of their targets are denoted as shown in Figure 3. The association of relationships of targets are indicate by lines, which connect two targets. The results of unnormalized OSPA, normalized OSPA, GOSPA, and OSPA, plus the miss-classification penalty and OSPA extended with the HMC label metric are recorded in Table 2.

As shown in Figure 3a, since the traditional OSPA-type metrics aim at finite sets, no categorical labels are taken into account. Therefore, the association step of OSPA only considers the localization bias distance, regardless of the label metrics. This leads to incorrect or cross error association in some cases. In these cases, directly adding the penalty for miss-classification will cause a counterintuitive high penalty, as highlighted in Table 2. In this paper, a label metric for the HMC labels is proposed. The results shown by Figure 3b illustrated that the mathematical consistent single metric solves the above issues by combining both the target state estimation bias and identity-aware confusion penalty into account.

## 5. Conclusions

The paper concerns on metrics of finite sets. Aiming at multiple quantities and types of targets, multi-class multi-target tracking usually faces not only cardinality errors, but also mis-classification problems. Considering its performance evaluation, the traditional optimal subpattern assignment (OSPA) method tends to calculate a separate metric for each class of targets, or introduce other indexes such as classification error rate, which decreases the value of OSPA as a comprehensive single metric.

This paper proposed a hierarchical multi-level class label with its metric for multi-class multi-target tracking under hierarchical multilevel classification, which can synthetically measure state errors, cardinality error, and mis-classification. Two contributions then can be summarized: (1) One kind of multilevel class label based on hierarchical tree structured category has been introduced, which enhanced finite sets with a label that can completely cover the space of multi-class multi-target tracking problems. (2) A mathematically metric of aforementioned hierarchical multilevel class label has also been given by extending the traditional OSPA metric as a new metric of finite sets with hierarchical multilevel class label, which can serve as the foundation for later researches on tracking algorithms.

The follow-up work includes extending the OSPA metric as a practical metric in the performance evaluation of multi-class multi-target tracking, studying the sensors management for multi-class multi-target tracking, and so on.

## Figures and Tables

**Table 1:** The label metric generates 15 pairs of distance between HMC labels. (Abbreviations: Ped—Pedestrian, Cyc—Cyclist, Veh—Vehicle, and Tar—Target).

Ped Cyc	Ped Car	Ped Van	Ped Veh	Ped Tar	Cyc Car	Cyc Van	Cyc Veh	Cyc Tar	Car Van	Car Veh	Car Tar	Van Veh	Van Tar	Veh Tar
5.00	8.60	9.43	8.72	7.10	7.00	8.00	7.14	5.50	1.00	0.14	2.36	0.86	3.14	2.30

**Table 2:** The numerical results of unnormalized OSPA, normalized OSPA, GOSPA, OSPA plus miss-classification penalty and OSPA extended with HMC label metric.

Unnormalized OSPA	Normalized OSPA	GOSPA	OSPA + Penalty	OSPA with HMC
5.2154	2.3324	3.8341	**27.6981**	**6.5415**

## Abbreviations

The following abbreviations are used in this manuscript:

MTT	Multi-target tracking
STT	Single-target tracking
OMAT	Optimal mass transfer
OSPA	Optimal subpattern assignment
GOSPA	Generalized OSPA
HMC	Hierarchical multilevel classification
FOV	Field of view
V2X	Vehicle-to-everything
SAR	Synthetic aperture radar
AIS	Automatic identification system
